# The roles of long non-coding RNAs in ovarian cancer: from functions to therapeutic implications

**DOI:** 10.3389/fonc.2024.1332528

**Published:** 2024-04-25

**Authors:** Zhong Hu, Lijin Yuan, Xiu Yang, Cunjian Yi, Jinzhi Lu

**Affiliations:** ^1^Department of Obstetrics and Gynecology, The First Affiliated Hospital of Yangtze University, Jingzhou, Hubei, China; ^2^Department of Obstetrics and Gynecology, Huangshi Puren Hospital, Huangshi, Hubei, China; ^3^Department of Obstetrics and Gynecology, Huangshi Central Hospital, Huangshi, Hubei, China; ^4^Department of Laboratory Medicine, The First Affiliated Hospital of Yangtze University, Jingzhou, Hubei, China

**Keywords:** long non-coding RNAs, ovarian cancer, biomarker, diagnosis, cancer therapy

## Abstract

Long non-coding RNAs (lncRNAs) are multifunctional and participate in a variety of biological processes and gene regulatory networks. The deregulation of lncRNAs has been extensively implicated in diverse human diseases, especially in cancers. Overwhelming evidence demonstrates that lncRNAs are essential to the pathophysiological processes of ovarian cancer (OC), acting as regulators involved in metastasis, cell death, chemoresistance, and tumor immunity. In this review, we illustrate the expanded functions of lncRNAs in the initiation and progression of OC and elaborate on the signaling pathways in which they pitch. Additionally, the potential clinical applications of lncRNAs as biomarkers in the diagnosis and treatment of OC were emphasized, cementing the bridge of communication between clinical practice and basic research.

## 1 Introduction

Ovarian cancer (OC), a silent killer, has become the second most prevalent gynecologic malignancy with the highest mortality rate worldwide (1). About 13,900 people in the U.S. reportedly died from this tumor in 2010, and fewer than 40 percent of female patients are cured (2). Due to its asymptomatic character, approximately 70% of patients are diagnosed at an advanced stage accompanied by tumor metastasis to the peritoneum (3, 4). Lumpectomy followed by platinum/paclitaxel-based pharmacotherapy is considered to be the standard treatment for OC (5). However, chemotherapeutic resistance is highly prevalent, ultimately leading to tumor recurrence and adverse prognosis. Despite an initial response to chemotherapeutic agents, the five-year survival rate of OC patients remains low due to chemoresistance (6). A significant barrier in the clinical management of OC has been reported to be the lack of ideal drug resistance-related biomarkers to delineate risk and determine prognosis (7). Moreover, effective screening program to detect OC at a curable early stage has faced great difficulties within recent years (4). With a survival rate of approximately 29%, these patients diagnosed at FIGO (Federation of International of Gynecologists and Obstetricians) stage III-IV are in dire need of a more effective treatment option (8). Confronted with these challenges, it’s crucial and critical to identify effective biomarkers for early diagnosis and therapy to improve survival in this aggressive disease.

Long non-coding RNAs (lncRNAs), which are over 200 nucleotides in length, have emerged as a dominant group of non-coding RNA molecules involved in diverse biological processes (9). Catalyzed by RNA sequencing, epigenomic and predictive technologies, a plethora of lncRNAs have been identified (10). Numerous researches have validated that abnormal expressions of lncRNAs are associated with a number of important pathophysiological processes in various diseases, including tumorigenesis (11–13). In particular, cumulative evidences have identified that aberrant expression of lncRNAs can serve as notable regulators in the initiation and development of OC, involving diverse molecular mechanisms. For instance, lncRNA-H19 interacts with miR-29b-3p and inhibits its downstream target gene STAT3, leading to carboplatin resistance in OC (14). In addition, lncRNA FLVCR1-AS1 regulates the miR-513/YAP1 axis and contributes to cell migration, invasion and epithelial mesenchymal transition (EMT) processes in OC (15). These studies imply that lncRNAs have great potential as therapeutic targets for OC patients.

We retrieved 1150 articles from 2000 to 2024 from the “GeenMedical” website under the keywords “lncRNA” and “ovarian cancer”, and a total of 131 preclinical and biological research, 41 review articles and 5 epidemiological studies were rigorously chosen in this review. This article describes the recognized functions and molecular mechanisms of lncRNAs and highlights their prospect as potential diagnostic, prognostic and therapeutic targets in OC.

## 2 Overview of LncRNA

### 2.1 Characteristics of LncRNA

LncRNAs are historically transcribed by RNA polymerase II from the genome and have lower expression levels than mRNA (16, 17). Additionally, lncRNAs are modified with capping and polyadenylation which makes majority of them stable, maintaining their presence in the cellular environment and facilitating the regulation of various cellular functions (18–20). With respect to genes coding proteins, lncRNAs can exist in intergenic, intronic or antisense regions (21). Although, initially, the synthesis of some lncRNAs was dismissed as merely transcriptional “noise” due to their commonly low levels of sequence conservation and expression (22). While, studies have shown that lncRNAs have dynamic expression patterns. For example, during development, lncRNAs gradually transition from widely expressed and conserved lncRNAs to cell lineage- and organ-specific lncRNAs (23). In addition, a recent study has shown that low abundance levels of lncRNAs are essential for their functional roles (24).

Intriguingly, cytoplasmic lncRNAs are less abundant and more stable relative to the nucleus (25). Importantly, nuclear lncRNAs regulate gene transcription by interacting with chromatin, whereas cytoplasmic lncRNAs induce biological signaling and post-transcriptional regulations (26). Moreover, it has been proved that lncRNA localization is associated with diverse pathophysiological statuses, including diseases such as various cancers (27). One study demonstrated that lncRNA TPT1‐AS1 was overexpressed in metastatic OC tissues and localized in the nucleus (28). Additionally, cell-specific and tissue-specific expression patterns are distinctive features of lncRNAs (29), facilitating the identification of lesions in specific tissues and the classification of different cell types (30, 31). Surprisingly, there was an unexpected specificity between dysregulated lncRNAs and histological isoforms of OC (32). Therefore, the exploration of the tissue specificity of lncRNAs will help to discover new markers associated with cancer types and apply them to the early detection of tumors or anti-cancer therapy.

### 2.2 Mechanisms of LncRNA functions

Mounting independent studies have shown that the distinctive feature of lncRNAs is their extensive incorporation of numerous molecules including DNAs, proteins, and RNAs, which are accessible to drive various important tumor phenotypes and influence OC progression (33). Currently, based on the functional mechanisms classification, lncRNAs can be commonly categorized into 4 types: signal, decoy, guide and scaffold. From the evolutionary perspective, this implies an incremental modification process between the prototypes and changes the lncRNA function step by step (16).

**Figure 1:**
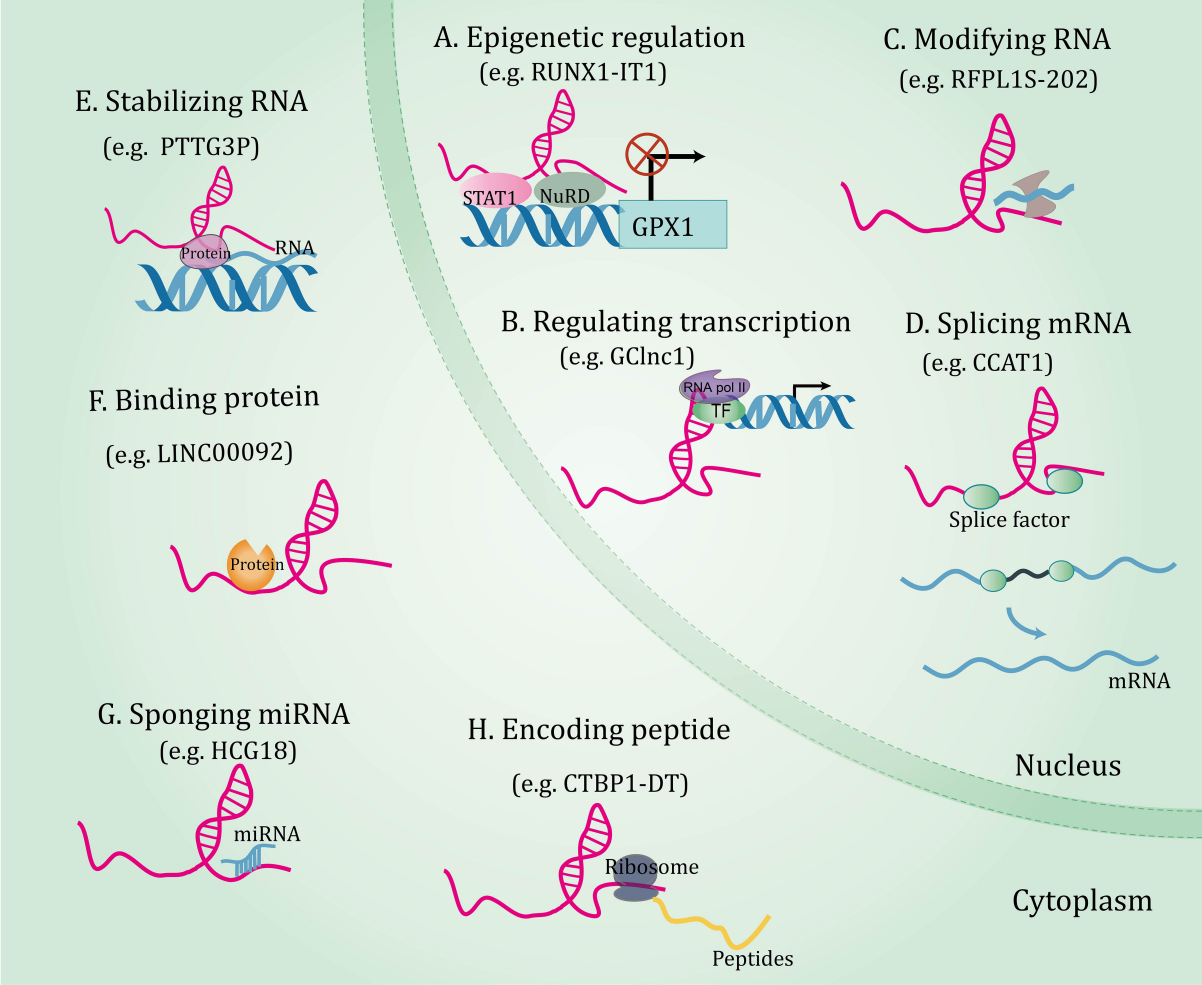
The functional mechanisms of long non-coding RNAs. Long non-coding RNAs orchestrate gene expressions through through multiple mechanisms, including epigenetic regulation **(A)**, regulating transcription **(B)**, and post-transcriptional regulations involving RNA modification **(C)**, splicing mRNA **(D)**, stabilizing RNA **(E)**, binding protein **(F)**, sponging miRNA **(G)**, and coding peptide **(H)**.

Decades of extensive research have elucidated the central role of lncRNAs in gene expression, laying the foundation for lncRNAs research more broadly. LncRNAs orchestrate gene expressions and exert a series of biological functions in disease through different mechanisms, which will be expounded in subsequent aspects (**Figure 1**). (I) On the epigenetic level, lncRNAs can recruit several epigenetic modifiers or related enzymes to affect gene expression (34, 35). (II) As transcriptional regulators, lncRNAs can bind to downstream gene promoters (36), transcription factors (37, 38), or recruit complexes to regulate gene expression patterns (39). (III) At the post-transcriptional level, lncRNAs interact with splicing factors to splice precursor mRNAs (40), combine with RNA-binding protein and affect mRNA stability (41, 42), and bind to proteins or mRNAs and impact their biological functions (43, 44). (IV) LncRNA acting as microRNAs (miRNAs) sponges to regulate the expression of their targets (45, 46). (V) Interestingly, a few lncRNAs bind to ribosomes and translate peptide products (47).

In summary, lncRNAs coordinate expressions of genes at the epigenetic, transcriptional, and post-transcriptional levels by interacting with a range of molecules, highlighting the fact that a comprehensive dissection of lncRNA-molecule interactions is key to understanding their functions. More detailed and comprehensive multi-species studies will help to discover more novel functions of lncRNAs.

### 2.3 Novel players in tumorgenesis

The widespread use of genetic databases and development of sequencing techniques have made it possible to functionally identify and characterize plenty of lncRNAs, triggering increased research in multiple diseases. Nowadays, lncRNAs have become a hotspot in the field of tumor research and are considered to play an essential role in the procession of OC. To date, whole transcriptome approaches such as expression microarrays and RNA sequencing have demonstrated that a number of lncRNAs are highly correlated with clinical outcomes in patients with OC. For instance, lncRNA HOTAIR was significantly upregulated in OC tissues and indicated poor clinical stage and prognosis in patients (48). Another well-described study demonstrated that lncRNA CCAT1 was related with tumor node metastases, histological grade, and FIGO stage of patients. In addition, high levels of CCAT1 were found to be an independent risk indicator for poor overall survival (OS) in patients (49). In contrast, some lncRNAs have been identified as critical suppressors of tumor progression in OC. For instance, lncRNA SLC25A21-AS1, LIMT and GAS5 inhibit tumor growth in OC through diverse mechanisms and signaling pathways (50–52). These findings reveal an important regulatory relationship between lncRNA and OC, although the in-depth pathogenesis of OC still remains to be explored. Therefore, analyzing the differential expression of lncRNAs in OC, combined with clinical information of patients and differences between cohorts, is beneficial to facilitate the discovery of new markers and the development of precision medicine. Going forward, they may have clinical applications in the appropriate management of OC patients.

## 3 Emerging roles of lncRNA in ovarian cancer biology

**Figure 2:**
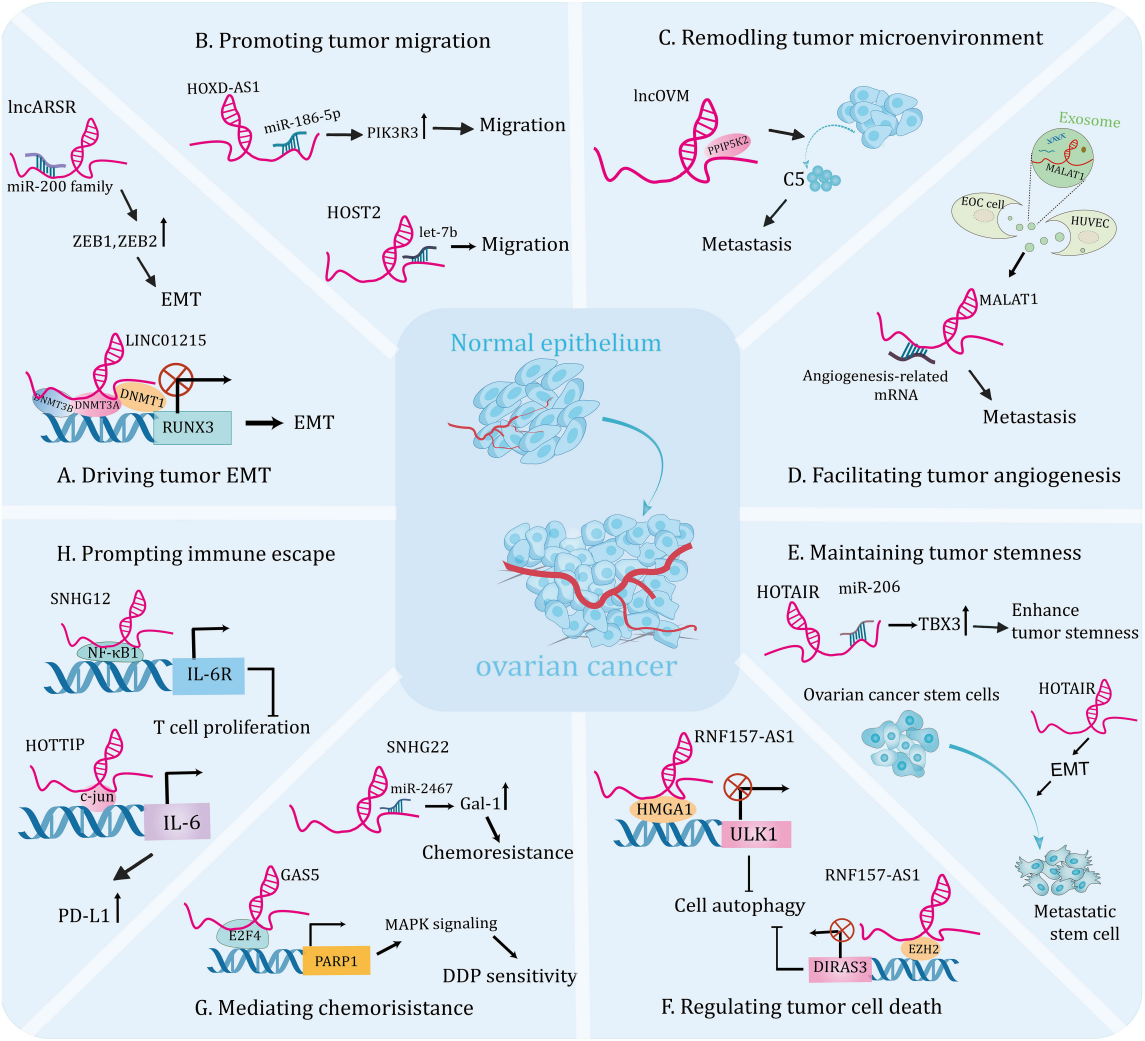
The emerging roles of lncRNAsin the tumorigenesis and progression of ovarian cancer. Molecular crosstalk pathways involving lncRNAs participated in ovarian cancer development, including EMT **(A)**, migration **(B)**, tumor microenvironment **(C)**, angiogenesis **(D)**, tumor stemness **(E)**, cell death **(F)**, chemoresistance **(G)**, and immune escape **(H)**.

With the advancement of science and technology, evidence for the association of dysregulated lncRNAs with OC is rapidly increasing. Through interaction with numerous molecules, including DNAs, proteins, and RNAs (53), lncRNAs are involved in the regulation of EMT, migration, tumor microenvironment (TME), angiogenesis, cancer stem cells (CSCs), apoptosis, autophagy, ferroptosis, chemoresistance, and tumor immunity, becoming important participants in the initiation and progression of OC. In the subsequent chapters, we will comprehensively sort out the functions and mechanisms that lncRNAs continue to be unearthed in OC genesis and development (**Table 1** and **Figure 2**).

**Table 1:** Functions of Long non-coding RNAs involved in ovarian cancer.

LncRNAs	Interactions	Expression	Molecular Mechanisms	Functions	Ref.
LncRNAs and tumor metastasis					
RUNX1-IT1	RNA-protein	Up	RUNX1-IT1/HDAC1 & STAT1/GPX1/NF-κB	Facilitates EMT and metastasis	(54)
KCNQ1OT1	RNA-protein	Up	KCNQ1OT1/EIF2B5	Drives tumor growth and metastasis	(55)
LINC01215	RNA-protein	Up	LINC01215/RUNX3	Promotes epithelial mesenchymal transition (EMT) and migration	(35)
PTAR	RNA-RNA	Up	PTAR/miR-101/ZEB1	Facilitates EMT, migration and metastasis	(56)
lncARSR	RNA-RNA	Up	lncARSR/miR-200family/ZEB1/ZEB2	Promotes EMT process, proliferation and invasion	(57)
HOXD-AS1	RNA-RNA	Up	HOXD-AS1/miR-186-5p/PIK3R3	Enhances EMT, migration and invasion	(45)
SNHG1	RNA-RNA	Up	SNHG/miR‐454/ZEB1	Facilitate cell migration, EMT, and invasion	(38)
HCG18	RNA-RNA	Up	HCG18/miR-29a/b/TRAF4 & TRAF5	Induces EMT and invasion	(58)
HOST2	RNA-RNA	Up	HOST2 /let-7b	Prompts proliferation, migration and EMT	(46)
CASC15	RNA-RNA	Up	CASC15/miR-23b-3p & miR-24-3p/SMAD3	Promotes tumor metastasis	(59)
UCA1	RNA-RNA	Up	UCA1/miR-485-5p/MMP14	Enhances metastasis, prognostic biomarker	(60)
ABHD11-AS1	RNA-protein	Up	ABHD11-AS1/RhoC/P70s6k & MMP2 & BCL-xl	Promotes proliferation, invasion, and metastasis, inhibits apoptosis	(61)
TC0101441	RNA-protein	Up	TC0101441/KiSS1	Promotes EMT and metastasis	(62)
LINC00092	RNA-protein	Up	LINC00092/PFKFB2	Drives glycolysis to induce metastasis	(63)
MALAT1	--	Up	--	Facilitates chemoresistance and invasiveness in TME	(64)
lncOVM	RNA-protein	Up	lncOVM/PPIP5K2/C5	Prompts tumorigenesis and metastasis	(65)
TMPO-AS1	RNA-protein	Up	TMPO-AS1/E2F6/LCN2	Facilitates aggressiveness and angiogenesis	(66)
DANCR	RNA-RNA	Up	DANCR/miR‐145/VEGF	Drives tumor angiogenesis and growth	(67)
MALAT1	RNA-RNA	Up	MALAT1/angiogenesis-related mRNAs	Promotes angiogenesis and metastasis	(68)
ATB	RNA-RNA	Up	ATB/miR-204-3p/TGFβR2	Prompts angiogenesis and remodels TME	(69)
HOTAIR	RNA-RNA	Up	HOTAIR/miR-206/TBX3	Maintains tumor stemness	(70)
LncRNAs and cell death					
HULC	RNA-protein	Up	HULC/ATG7	Promotes tumor growth, inhibit autophagy, diagnostic marker	(71)
Meg3	RNA-protein	Down	Meg3/ATG3	Inhibits autophagy and tumorigenesis	(72)
RNF157-AS1	RNA-protein	Up	RNF157-AS1/HMGA1 & EZH2/ULK1 & DIRAS3	Represses autophagy to enhance viability in good environment, while inhibits tumor growth in harsh environment	(36)
RP11-552M11.4	RNA-DNA	Up	RP11-552M11.4/BRCA2	Promotes proliferation, migration, and invasion	(73)
ANRIL	RNA-protein	Up	ANRIL/P15INK4B & Bcl-2	Promotes proliferation and cell cycle progression , inhibits apoptosis	(74)
PCGEM1	RNA-protein	Up	PCGEM1/RhoA	Facilitates proliferation, migration, invasion, suppresses apoptosis	(75)
DLEU1	RNA-RNA	Up	DLEU1/miR‐490‐3p/CDK1& CCND1 & SMARCD1	Prompts tumor pathogenesis and development	(76)
CACNA1G-AS1	RNA-protein	Up	CACNA1G-AS1/IGF2BP1/FTH1	Inhibits ferroptosis, promotes proliferation and migration	(77)
ADAMTS9-AS1	RNA-RNA	Up	ADAMTS9-AS1/miR-587/ SLC7A11	Inhibits ferroptosis, promotes proliferation and migration	(78)
LncRNAs and drug resistance					
GAS5	RNA-protein	Down	GAS5/E2F4/PARP1/MAPK	Inhibits cisplatin resistance	(37)
ZFAS1	RNA-RNA	Up	ZFAS1/miR-150-5p/Sp1	Strengthens cisplatin and paclitaxel resistance	(79)
SNHG22	RNA-RNA	Up	SNHG22/miR-2467/Gal-1	Promotes cisplatin and paclitaxel resistance	(80)
CCAT1	RNA-RNA	Up	CCAT1/miR-454/survivin	Enhances cisplatin resistant	(81)
RFPL1S-202	RNA-protein	Down	RFPL1S-202/DDX3X/IFN-β-STAT1	Suppresses chemoresistance	(43)
MIR17HG	RNA-protein	Down	KHDRBS3/MIR17HG/CLDN6	Promotes paclitaxel resistance and induces glycolysis	(82)
PVT1	RNA-protein	Up	PVT1/JAK2/STAT3/PD-L1	Prompts cisplatin resistance	(83)
LncRNAs and tumor immunity					
HOTTIP	RNA-protein	Up	HOTTIP/c-jun/IL-6/PD-L1	Immune escape, suppresses activities of T cells	(84)
Xist	RNA-RNA	Up	Xist/miR-101/KLF6/C/EBPα	Attenuates M1 to M2 macrophages	(85)
SNHG12	RNA-protein	Up	SNHG12/NF-κB1/IL-6R	Suppresses T cell proliferation	(86)
CTD-2288O8.1	RNA-protein	Up	CTD-2288O8.1/EGFR/AKT	Promotes M2 macrophage polarization	(87)
ZFHX4-AS1	--	Up	--	Prompts proliferation, invasion and migration	(88)
PAXIP1-AS1	--	Down	--	lymphatic invasion	(89)

### 3.1 LncRNAs modulate tumor metastasis by regulating EMT, migration, TME, angiogenesis, and CSC

Tumor metastasis is a major cause of treatment failure and death in patients (90, 91). It is a complex process characterized by the spread of cancer cells from the primary tumor to distant organs by acquiring certain molecular and phenotypic changes (92). In recent years, mounting studies have shown that lncRNAs play a key role in EMT, migration, TME, angiogenesis, and CSC, thus influencing the metastatic cascade of OC. Therefore, exploring the mechanisms and signaling pathways involved in metastasis of OC will provide a basis for the development of anti-tumor drugs to improve the prognosis of female patients.

EMT refers to the endowment of epithelial cells with mesenchymal feature, allowing tumor cells to leave primary site of the tumor, then invading the paracancerous tissue and migrating to distant organs (93). Thereby, cancerous mesenchymal cells acquire enhanced motility and ability to invade the surrounding tissues (94, 95). Another core character of EMT is that cancer cells are capable of stem cell-like characteristics, allowing tumor cells relentlessly differentiate into numerous tumor cells (96). Therefore, activation of EMT under pathological condition will benefit for fostering tumor progression. Increasing independent researches have indicated that lncRNAs can induce EMT program (45) to affect biological behavior of tumor cells and correlate with cancer aggressiveness (33). Below are some examples of lncRNAs acting as regulators involved in tumor EMT activation and migration, thereby triggering OC metastasis through multiple pathways.

LncRNAs can affect EMT by interacting with epigenetic regulation-related factors to modulate gene expression, thereby inducing the initiation and progression of OC. For instance, one study identified that lncRNA RUNX1-IT1 was upregulated in metastatic OC samples and promoted the EMT and metastasis of cell lines. Mechanistically, RUNX1-IT1 serves as a molecular scaffold coupling the transcription factor STAT1 to the NuRD complex, colocalizes with the GPX1 promoter and promotes its deacetylation, epigenetically inhibits GPX1 transcription, thereby activating the NF-κB pathway and driving ovarian malignancy (54). Furthermore, DNA methylation has been shown to be a hallmark of human cancers and is essential for gene expression (97, 98). A recent research revealed that the expression level of lncRNA KCNQ1OT1 was elevated in OC tissues and it’s knockdown inhibited EMT and metastasis of tumor cells. Mechanistically, KCNQ1OT1 recruits DNA methyltransferases to the EIF2B5 promoter, resulting in decreased EIF2B5 expression, which contributes to the development of OC (55). In addition, another research demonstrated that the expression of lncRNA LINC01215 was significantly upregulated in OC tissues compared to the normal tissues. Further functional experiment uncovered that silencing LINC01215 inhibited EMT, thus impairing tumor growth and metastasis. Mechanistically, nuclear LINC01215 recruited methylation-related proteins (DNMT1, DNMT3A, and DNMT3B) to prompt methylation of the RUNX3 promoter, thereby reducing RUNX3 expression. In addition, overexpression of LINC01215 resulted in significant acceleration of tumor growth, enlarged tumor volume, and increased metastasis observed in tumor xenograft models (**Figure 2A**) (35).

The miRNA molecule is a single-stranded RNA that modulates gene expression by leading to degradation, denaturation, or translational inhibition of targeted mRNA (99). An increasing number of studies indicate that the interaction between lncRNA and miRNA plays a crucial role in the progression and metastasis of OC. For instance, lncRNA PTAR promoted EMT and metastasis of OC cells by competitively interacting with miR-101 to elevate the expression of ZEB1. Furthermore, knockdown of PTAR attenuated tumor growth and metastasis *in vivo* (56). A recent study showed that lncARSR was highly expressed in OC tissues and was linked to lymph node metastasis. Mechanistically, lncARSR interacted with miR-200 family to increase the expression of ZEB1 and ZEB2, resulting in an upregulation of N-cadherin and a downregulation of E-cadherin and thus promoting EMT progression. Moreover, lncARSR could increase β-catenin expression via interacting with HuR, triggering Wnt/β-catenin pathway to promote cell proliferation (**Figure 2A**) (57). Another study indicated that upregulation of lncRNA HOXD-AS1 in OC tissues was associated with high tumor grade, advanced stage and lymph node metastasis in patients. Thus, it could be the independent risk predictor for OC patients. Further analysis uncovered that knockdown of HOXD-AS1 significantly reduced the ability of migration, invasion and EMT. Mechanistically, HOXD-AS1 sponges miR-186-5p to increase the expression of PIK3R3, thus facilitating tumor development (**Figure 2B**) (45). In addition, Wu et al. showed that lncRNA SNHG1 was overexpressed in OC and could facilitate cell migration, EMT, and invasion of tumor cells *in vitro*, as well as enhance metastasis *in vivo*. Mechanistically, SNHG drives tumor progression by interacting with miR‐454 to increase ZEB1 expression, activating the Akt signaling pathway (100). Similarly, high expression of lncRNA HCG18 was detected in OC tissues and it drove EMT, proliferation and migration of cell lines by modulating the miR-29a/b/TRAF4/TRAF5 axis (58). Interestingly, a recent study showed that OC-specific lncRNA HOST2 could sponge let-7b to promote the endogenous expressions of oncogenes, facilitating tumor cell migration, invasion, and proliferation (**Figure 2B**) (46). Lin et al. reported that lncRNA CASC15 is an oncogene of OC that promotes tumor metastasis through a TGF-β-induced EMT program (59). Additionally, high expression of lncRNA UCA1 in OC cells and tissues was significantly linked to lymphatic metastasis of the tumor. Further analysis revealed that UCA1 knockdown significantly inhibited the invasive and migratory abilities of tumor cell lines. Mechanistically, UCA1 could increase the expression of MMP14 through sponging miR-485-5p, thereby promoting tumor metastasis. Therefore, the UCA1/miR-485-5p/MMP14 axis may provide a potential strategy for targeting tumor metastasis-related therapies (60).

In addition, lncRNAs can promote metastasis or maintain tumor biological functions by regulating the expression of oncogenes or EMT-related genes (101, 102). For instance, one study found that high expression of lncRNA ABHD11-AS1 in OC tissues could promote tumor metastasis *in vitro* and enhance intraperitoneal metastasis of tumors *in vivo*, and was positively correlated with tumor stage, while was inversely proportional to the degree of tumor differentiation. Further trial verified that ABHD11-AS1 could interact with RhoC and target its downstream molecules including matrix metalloprotease 2 (MMP2), promoting tumor growth (61). As known to us, RhoC is involved in regulating cell adhesion and migration ability. MMP2, as an oncogenic genes, plays an important role in the invasion and metastasis of cancer cells (103). Another study observed that the expression of lncRNA TC0101441 was highly elevated in OC tissues compared with noncancerous tissues and was significantly associated with advanced FIGO stage and poor prognosis. Functional assays showed that TC0101441 knockdown resulted in elevated E-cadherin levels and downregulation of N-cadherin in mouse tumor tissues compared to controls. Mechanistically, TC0101441 downregulated its downstream gene KiSS1, which enhanced EMT-induced invasion (62). While, another independent study showed that lncRNA AOC4P exerts anti-metastatic effects in OC and was negatively correlated with advanced tumor stage and lymphatic metastasis. *In vitro* experiments confirmed that expression of AOC4P reduced the metastatic ability of highly metastatic tumor cells, which was also demonstrated in an intraperitoneal metastatic model. Mechanistically, AOC4P inhibited the expression of EMT-related genes MMP9 and COL1A2, thereby attenuating invasive metastasis of OC (104). Similarly, high expression of lncRNA HOTAIR was observed in OC tissues and metastatic cells than in parental cells. Further experiments suggested that HOTAIR regulated matrix metalloproteinases and EMT-related genes, facilitating migration and invasion of cell lines and promoting intraperitoneal tumor growth and metastasis in nude mice. Notably, HOTAIR could be independently linked to disease-free survival and OS in patients (48).

Taken together, the above researches have identified the extensive and key roles of lncRNAs in OC metastasis through initiating EMT and migration. Therefore, targeting these lncRNAs could be a potential strategy for the treatment of this neoplasms. Consequently, based on these anticipations, further and systematic research on lncRNAs is extremely necessary which will help us grasp the detailed mechanisms of their actions in more *in vivo* models, providing us with guidance for future practice.

Mounting studies have unraveled that TME is a complex ecosystem with diverse tumor cells, stroma cells, exosomes and other components, which is crucial for facilitating tumor metastasis and invasion through the signaling in the communication circuits established by these ingredients (105). Cancer-associated fibroblasts (CAFs) have been shown to participate in the stromal-dependent alterations that contribute to the initiation and progression of malignant neoplasms (106). One study showed that the metastatic factor CXCL14, secreted by CAFs, induced high expression of lncRNA LINC00092, which contributed to invasion and migration of OC cells. Further analysis demonstrated that silence of LINC00092 significantly reduced ascites and metastatic nodules in *in vivo* models compared with the controls. Mechanistically, LINC00092 can interact with PFKFB2 to promote metastasis by promoting glycolysis, which is essential for the malignant characterization of TME and the maintenance of functions of CAFs. Moreover, high levels of LINC00092 suggested poor OS, advanced stage, higher differentiation degree and more resistant disease in patients (63). This hints an inseparable connection between TME and tumor metastasis. Similarly, another research indicated that lncRNA MALAT1 could also promote metastasis and invasiveness of OC through affecting TME. Further investigation found that overexpression of MALAT1 resulted in increased vimentin and cytokine interleukin-1β, indicating stimulation of an inflammatory response in TME, thereby triggering OC progression. In addition, simultaneous overexpression of MALAT1 in OC cells and cancer-associated fibroblasts greatly enhanced the invasion of OC cells (64). Interestingly, lncRNAs can modulate immune cell infiltration into the tumor microenvironment and enhance metastasis of OC. For instance, lncOVM can interact with protein PPIP5K2 and protect it from degradation, leading to an increase in complement C5 secretion by tumor cells, which in turn recruits myeloid-derived suppressor cells to infiltrate the TME and promote OC metastasis. Furthermore, this research suggests that lncOVM may serve as a potential marker for the early diagnosis of OC (**Figure 2C**) (65).

Growing evidence has shown that angiogenesis is a prerequisite for metastasis and can help malignant cells leave the site of origin (107, 108). Numerous studies have shown that lncRNAs play an important role in tumor angiogenesis and influence tumor progression (109, 110). In addition, breakthroughs in the regulation of angiogenesis by lncRNAs have been made in studies of ovarian cancer. For instance, lncRNA TMPO-AS1 was upregulated in ovarian cancer samples and cell lines and was significantly associated with tumor angiogenesis and metastasis. Further experiments showed that both the aggressiveness of tumor cells and their pro-angiogenic capability were reduced by silencing it. Mechanistically, lncRNA TMPO-AS1 interacts with the transcription factor E2F6 to promote the transcription of lipocalin-2, thereby facilitating the progression of ovarian cancer (66). Likely, Lin et al. revealed that lncRNA DANCR drives tumor angiogenesis and growth through directly binding to miR‐145, thereby prompting VEGF expression in OC (67). Additionally, exosomes can mediate crosstalk between different cells during cancer progression by transporting their cargos, including lncRNA (30). Recent studies have identified a link between dysregulated expression of exosome-derived lncRNAs and tumor angiogenesis. For instance, the expression level of lncRNA MALAT1 was increased both in metastatic OC cell lines and in the exosomes they secreted. Functional experiments demonstrated that knockdown of MALAT1 can inhibit angiogenesis ability of human umbilical vein endothelial cells (HUVECs) induced by exosomes. In summary, MALAT1 was delivered to HUVECs by exosomes, triggering expressions of angiogenesis related genes. Importantly, elevated serum MALAT1 levels in patients are strongly correlated with advanced OC and metastasis in clinical settings (**Figure 2D**) (68). Likely, another study showed that exosomal lncRNA ATB secreted by OC cells could be transferred to HUVECs and promote their angiogenesis, which in turn promotes tumorigenesis by remodeling the TME via modulation of the miR-204-3p/TGFβR2 signaling pathway (69). Thus, attenuation of specific lncRNAs in tumor cell-derived exosomes will be instrumental in inhibiting tumor growth and metastasis. However, further preclinical studies are needed to validate the feasibility of this theoretical scenario.

CSCs exhibit high proliferative and invasive activities and are resistant to a wide range of chemotherapeutic drugs, which are important causes of tumor metastasis (111). Targeting CSCs may lead to the discovery of efficacious approaches to eradicate the malignant tumor (112). Emerging evidence supports that lncRNAs play a vital role in regulating CSCs, therefore affecting the progression of OC (113). A recent study demonstrated that the expression of lncRNA HOTAIR was significantly increased in the OC patient’s tumor tissues and OC stem cells compared with controls. Further investigation showed that down-regulated HOTAIR in stem cells suppressed the tumor growth and lung metastasis in the nude mouse model (**Figure 2E**) (114). Interestingly, another independent study showed that HOTAIR upregulated TBX3 expression by binding to miR-206, thereby maintaining the stemness of OC stem cells (**Figure 2E**) (70). This research reveals that targeting lncRNA HOTAIR in stem cells could be a promising therapeutic strategy for therapy of OC patients. Indeed, certain inhibitors may target CSCs by modulating lncRNAs to exert anti-tumor effects. A recent study showed that the expression of lncRNA-Meg3 was significantly decreased in anisomycin-treated serous ovarian CSCs. Further experiment demonstrated that anisomycin was effective in inhibiting the angiogenic capacity of ovarian CSCs *in vitro* and *in vivo*. Mechanistically, anisomycin targets the lncRNA-Meg3/miR-421/PDGFRA axis, thereby inhibiting the malignant behavior and angiogenic capacity of CSCs (115). In addition, isomycin was shown to inhibit the proliferation, invasion and tumorigenesis of ovarian CSCs by increasing the expression of lncRNA BACE1-AS (116). This represents a theoretical basis for the development of new drugs to OC.

Here, we retrospect how lncRNAs promote tumor metastasis by influencing the TME, angiogenesis, CSCs through different mechanisms, and by certain genes in the pro-metastatic step. Therefore, lncRNAs may be potential targets for the treatment of OC metastasis, and it is warranted to further study lncRNAs to deepen our understanding of their mechanisms of actions in various *in vivo* models, advancing their potential clinical applications.

### 3.2 LncRNAs regulate cell death

Cell homeostasis plays an important role in maintaining a healthy cellular environment and it’s imbalance may lead to the occurrence of cancer (71). Studies have shown that regulated cell death (RCD) is involved in sustaining cell homeostasis and the dysregulation of pathways can lead to the developments of cancer (117). In recent years, the induction of RCD in tumor cells has been a therapeutic strategy, including programs related to autophagy, apoptosis, and ferroptosis (118). Therefore, it is crucial to understand the underlying molecular mechanisms of RCD in OC. Here, we outline the regulatory relationships between lncRNAs and RCDs (autophagy, apoptosis, and ferroptosis) in OC, which is expected to provide new ideas for the diagnosis and treatment of this disease.

Autophagy, an intracellular lysosomal degradation pathway, functions to degrade and recycle excess or damaged cells (119). It has been proved to play pivotal roles in promoting death and apoptosis of tumor cells and responsible for tumor growth (89, 120). One study reported that the expression level of lncRNA HULC were significantly elevated in OC tissues, and it could interact with the autophagic protein ATG7 and reduce its expression, promoting tumor growth through. Further assay revealed that silence of HULC could boost apoptosis and inhibit cell proliferation, which could be reversed by autophagy inhibitors. Furthermore, HULC could facilitate OC progression through regulating ITGB1 (71). While, another research showed that lncRNA Meg3 was lowly expressed in OC tissue and was negatively correlated with FIGO stages. Further studies demonstrated that Meg3 interacted with ATG3 protein and increased its expression, which significantly increased apoptosis in cell lines and mediated their arrest in G2 phase, as well as attenuated tumorigenesis *in vivo* (72). However, autophagy is a double-edged sword based on its impacts on cell survival under different circumstances. One research indicated that overexpression of lncRNA RNF157-AS1 in a favorable environment could promote the resistance of OC cells to cisplatin (DDP) by mediating autophagy. While, under the DDP treatment, overexpression of RNF157-AS1 could decrease autophagy, promote apoptosis and sensitivity of cell lines to chemotherapy, and reduce tumor size and weight in mice. On balance, RNF157-AS1 recruits EZH2 and HMGA1 to bind the DIRAS3 and ULK1 promoters, respectively, thereby inhibiting transcriptions of these two autophagy-related genes. Moreover, patients with high expression of RNF157-AS1 had better prognosis than those with low expressions, so it could be used as a prognostic indicator for OC patients (**Figure 2F**) (36).

It’s of no doubt that apoptosis is a regulated cell death mechanism that is important in majority of physiological processes, such as the development process and tumorigenesis (121, 122). Studies have demonstrated that apoptosis inhibitors play an oncogenic role in a variety of tumors (123, 124). Therefore, understanding the involvement of lncRNAs in the regulation of apoptosis in OC will be beneficial for exploring new relevant therapeutic approaches based on the promotion of apoptosis in tumor cells. Caspases have been shown to play a central regulatory role in apoptosis, once activated, lead to the cleavage of proteins in the cell, triggering apoptosis (125). Moreover, apoptosis is extensively regulated by the Bcl-2 protein and its family (126). For instance, the expression level of lncRNA RP11‐552M11.4 was elevated in OC tissue compared to paired non-tumor samples, and was correlated with pathological grade, huge tumor size(≥10 cm) and advanced FIGO stages (III‐IV). Furthermore, upregulation of RP11-552M11.4 enhanced the proliferation, migration, and invasion of cell lines, implying that it potentially turned normal ovarian epithelial cells into cancer-like cells. Further experiment indicated that exogenous overexpression of RP11‐552M11.4 suppressed cell apoptosis through targeting BRCA2 and attenuating its transcription, promoting tumor growth (73). In addition, one study showed that lncRNA ANRIL was highly expressed in OC samples and its overexpression could increase the expression of Bcl-2 protein, thus promoting cell proliferation and decreasing the number of apoptotic cells (74). Similarly, lncRNA PCGEM1 inhibited the apoptosis of OC cell by targeting RhoA and increasing its downstream BCL-xL protein expressions, promoting OC progression (75). Additionally, lncRNA DLEU1 could inhibit apoptosis and boost the development of OC by interacting with miR-490-3p to increase the expressions of Bcl‐xL proteins (76).

Some lncRNAs may modulate the other types of RCD in OC, including ferroptosis. Recently, a study confirmed that lncRNA CACNA1G-AS1 was obviously upregulated in OC samples. Further analysis uncovered that CACNA1G-AS1 could inhibit ferroptosis through increasing the expression of FTH1 via IGF2BP1 axis, thus promoting proliferation and migration in OC cells. In addition, *in vivo* studies revealed that knockdown of CACNA1G-AS1 could increase the sensitivities of OC cells to ferroptosis (77). Likely, another study demonstrated that lncRNA ADAMTS9-AS1 expression was elevated in OC cells. Further experiment showed that lncRNA ADAMTS9-AS1 inhibited ferroptosis by targeting miR-587 to promote the expression of SLC7A11, thereby facilitating the proliferation and migration of tumor (78).

In conclusion, above studies have shown that dysregulated lncRNAs trigger autophagy, apoptosis, and ferroptosis, thereby affecting the progression of OC, which suggests a new perspectives in the field of OC treatment. Therefore, conducting more researches on lncRNA in autophagy and apoptosis will help to better understand the important cellular processes involved and develop better therapeutic regimens to counteract the defects in autophagy and apoptosis associated with human tumors.

### 3.3 LncRNAs induce drug resistance

Resistance to chemotherapeutic drugs is considered to be the main cause of tumor treatment failures and disease recurrences (127). Moreover, the emergence of drug resistance seriously hampers the clinical application of chemotherapy and ultimately culminates in patient death (128). In recent years, the pivotal regulatory roles of lncRNAs in cancer drug resistance has received widespread attention. Several studies have demonstrated that lncRNAs critically contribute to the development of drug resistance in OC by modulating different signaling pathways or regulating the expressions of targeted genes involved in various cellular processes (79–81, 128). All these results have informed future chemosensitivity studies and provided new insights into the development of lncRNA-targeted drugs for clinical applications. The following section describes the current status of knowledge on the research into lncRNAs as regulators and predictors of OC resistance and emphasizes their underlying mechanisms of actions.

LncRNAs can interact with epigenetic regulators and affect gene transcription, thereby inducing chemoresistance and promoting tumor progression. For example, a study found that the dramatically down-regulated lncRNA GAS5 in OC tissues was associated with poor outcomes. Further analyses indicated that GAS5 overexpression enhanced the sensitivity of OC cells to DDP, and absence of GAS5 was more pronounced in drug-resistant cells compared to sensitive cells. Mechanistically, GAS5 recruits the transcription factor E2F4 to the PARP1 promoter and attenuates its transcription, thereby inhibiting the MAPK pathway and tumor progression. This study confirms that GAS5 regulates the E2F4/PARP1/MAPK axis and restrains OC progression. In addition, rapamycin could increase the expression of GAS5 in cytoplasm, providing a theoretical basis for the development of combination therapies for OC (**Figure 2G**) (37).

LncRNAs can also modulate cell biological behavior by acting as competing endogenous RNA for miRNAs, thereby affecting chemotherapy tolerance in OC. A recent study research demonstrated that the expression of lncRNA ZFAS1 was significantly elevated in OC tissues, especially in metastatic specimens. Functional experiments showed that ZFAS1 knockdown facilitated the sensitivity of cell lines to DDP and paclitaxel (PTX). Further analysis indicated that ZFAS1 promotes the expressions of the transcriptional factor Sp1 through sponging miR-150-5p, leading to chemoresistance and OC malignancy (79). Moreover, Zhang et al. revealed that lncRNA SNHG22 may be a potential prognostic biomarker and novel therapeutic target for OC. This is based on the fact that the expression level of SNHG22 is significantly upregulated in OC tissues and induces the miR-2467/Gal-1 axis, which promotes chemotherapy resistance (**Figure 2G**) (80). Another study elucidated that the expression of lncRNA CCAT1 was upregulated in DDP-resistant OC cell lines and knockdown of it restored cell sensitivity to DDP. Mechanistically, CCAT1 promotes chemoresistance through inhibiting cell apoptosis via the miR-454/surviving axis (81).

Moreover, lncRNA can affect chemoresistance through combining with proteins, thus influencing tumor progression. Liu et al. revealed that lncRNA RFPL1S-202 acted as a tumor suppressor during OC progression and overexpression of RFPL1S-202 enhanced the chemosensitivity of tumor cells to DDP or PTX, and impeded liver metastasis of tumor *in vivo* model. Mechanistically, RFPL1S-202 combines with DEAD-Box Helicase 3 X-linked protein and inhibits the IFN-β-STAT1 pathway, thereby suppressing chemoresistance (43). Recently, studies have suggested that enhanced aerobic glycolysis in the tumor environment may affect tumor tolerance to chemotherapeutic agents (129). Similarly, a study of OC resistance to chemotherapeutic agents showed that lncRNA MIR17HG was downregulated in cell lines and tissues based on the inhibitory effect of the RNA-binding protein named KHDRBS3. Further experiments showed that overexpression of KHDRBS3 promotes resistance to PTX in OC cells by enhancing glycolysis, which could be rescued by overexpression of MIR17HG (82). Therefore, the novel lncRNA-based combined glycolysis inhibitor against drug resistance of OC warrants further investigations. In addition, another study showed that overexpression of lncRNA PVT1 increased the invasive and proliferative capacity of DDP-resistant cells, suggesting more deteriorated clinical behaviors and poorer prognosis in OC patients. Further experiments showed that PVT1 prompts programmed cell death 1 ligand (PD-L1) expression by regulating the JAK2/STAT3 axis, thereby promoting tumor progression (83).

The aformentioned research studies indicate that lncRNAs can induce resistance to anticancer drugs in OC cells by interacting with DNA, miRNAs and proteins and modulating different pathways. Therefore, targeting drug resistance-associated lncRNAs may be a promising novel therapeutic approach to improve the prognosis of OC patients.

### 3.4 LncRNAs mediate tumor immunity

Tumor immune microenvironment (TIME) consists of a variety of immune cells and stromal components, creating a complex landscape that is important in tumor progression (130). The immune system plays an important role in regulating tumor cell death, harnessing these immune cells to battle the tumor cells could serve as potential targets for cancer therapy (131, 132). In terms of biological functions, lncRNAs are closely linked to tumor development as well as the establishment of a hostile TIME (8). Hence, identifying this key regulators in tumor immunity may provide potential candidates for therapeutic interventions. Recently, the multiple functions of lncRNAs in the regulation of immune cells, including neutrophils, T cells, and macrophages, have been emphasized in OC, providing evidence for their important role in tumor immunoregulation.

Neutrophils are the first line of host defense against infections and studies have shown that they respond to contact with cancer cells and have the potential to promote tumor progression (133). Besides, a previous study revealed that neutrophils could negatively regulate the adaptive immunity through PD-L1emerging as central players in tumor immune evasion (134, 135). Interestingly, multiple observations have exerted that lncRNAs could serve as crucial regulators of immune escape. A recent study showed that lncRNA HOTTIP was overexpressed in OC tissues. HOTTIP was noted to prompt the transcription of IL-6 by modulating c-jun, a transcription factor, consequently increased the expressions of PD-L1 in neutrophils, thereby suppressing activities of T cells and consequently potentiating the immune escape of OC cells, which uncovers a promising immunotherapeutic strategy by targeting HOTTIP in OC (**Figure 2H**) (84). Moreover, it was discovered that macrophages activated by cytokines have the ability to kill tumor cells (136). While, the skewing of macrophages towards the M2 phenotype creates an immunosuppressive microenvironment in the OC, which exerts a tumor-promoting effect (137). For instance, one study indicated that lncRNA Xist inhibited migration and proliferation of OC cell lines by altering the polarization of macrophages. Further experiment showed that Xist was upregulated in M1 macrophages and it’s knockdown induced M1 to M2 macrophages. Mechanistically, Xist inhibits the progression of OC by interacting with miR-101 to promote the expressions of Kruppel-like factor 6 and CCAAT/enhancer-binding protein (85). Recently, one study indicated that lncRNA SNHG12 was involved in immune escape by modulating the crosstalk between OC cells and M2 macrophages. Further studies showed that SNHG12 suppressed T cell proliferation by promoting the expressions of PD-L1 in both SKOV3 cells and M2 macrophages (**Figure 2H**) (86). Another study showed that lncRNA CTD-2288O8.1 was proved to accelerate the polarization of M2 macrophages, therefore facilitating immunosuppression of OC. Moreover, CTD-2288O8.1 was significantly related to the expression of PD-L1 and PD-L2, suggesting that it could predict the response to immunotherapy in OC patients (30). Another study showed that increased lncRNA ZFHX4-AS1 expressions were associated with poor OS in OC and further experiments confirmed that the surface marker CD206 of M2 macrophages was enriched in the high expression group (8). This result suggests that ZFHX4-AS1 is related with tumor-infiltrating immune cells and it can be served as a potential therapeutic target for OC in the future.

Nevertheless, other breakthroughs have been made in lncRNA-mediated immune infiltration in OC. For instance, lncRNA PAXIP1-AS1 was significantly downregulated in OC cell lines and its low expression level was significantly associated with poor survival and immune infiltration in OC. Moreover, gene set enrichment analysis demonstrated that neutrophil degranulation was differentially enriched in high expression phenotype of PAXIP1-AS1, which could be a promising response to immunotherapy for OC (138). Similarly, the expression of lncRNA LEMD1-AS1 was decreased in OC tissues compared to normal specimens, suggesting that patients has a shorter OS than those with high LEMD1-AS1 expression. Further analysis revealed that LEMD1-AS1 expressions were negatively correlated with the expressions of neutrophil cells. Overall, this finding hints that LEMD1-AS1 may have potential to be therapeutic target in epithelial ovarian cancer (EOC) (139). Interestingly, Huang et al. reported that lncRNA ZFHX4-AS1 was highly expressed in OC tissues and was negatively related with expression levels of T cell CD8+, neutrophil, and macrophage, suggesting that it may play a vital role in the immune microenvironment of OC (140).

Tumor resistance to immunotherapy has become a key factor affecting the efficacy of tumor therapy, which largely stems from the immunosuppressive properties of the tumor microenvironment (141). These studies provide a direction for understanding the role of lncRNAs in immunotherapy for OC. In the future, these studies on the role of lncRNAs in the TIME may help to provide more personalized immunotherapeutic interventions for OC patients.

## 4 Clinical relevance of lncRNAs in OC

**Figure 3:**
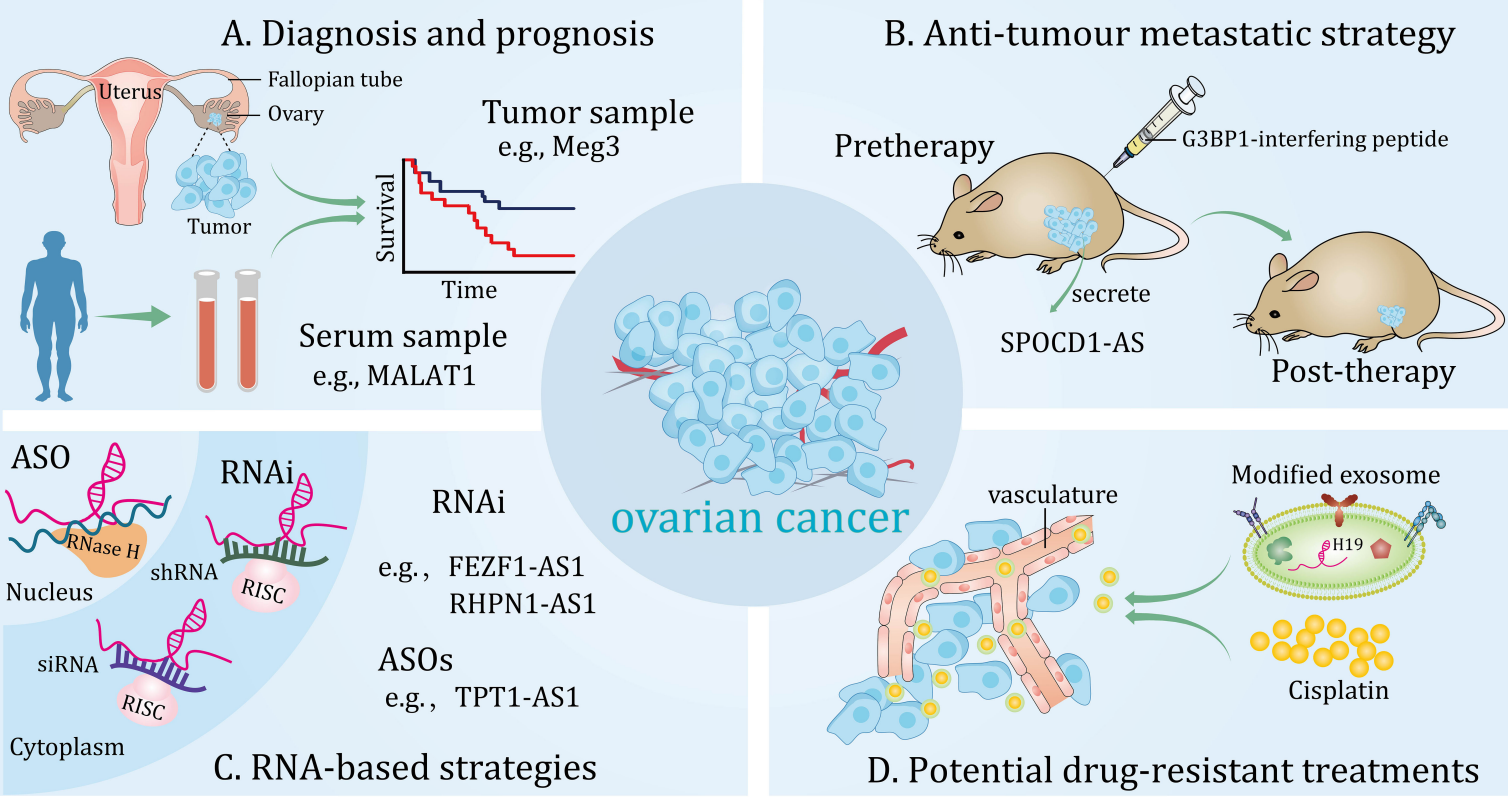
Potential clinical applications of long non-coding RNAs (lncRNAs) in ovarian cancer. These lncRNAs can be detected from different samples and are novel diagnostic and prognostic biomarkers **(A)**. Potential anti-tumour metastatic therapeutic strategy for targeting lncRNAs in *in vivo* models **(B)**. RNA-based strategies can effectively target lncRNAs in the cytoplasm and nucleus **(C)**. Modified exosomes loaded with cisplatin target ovarian cancer in vivo **(D)**.

Emerging studies have shown that the specific expression patterns of lncRNAs make them ideal candidates for valuable biomarkers to improve diagnostic efficiency. In addition, exosomal lncRNA secreted by cancer cells be effectively detected in several body fluids of human, including plasma, serum and saliva (142–144), hinting it may act as potential marker for diagnostic tests without invasive operations. More importantly, their functional potential has been validated in *in vivo* and *in vitro* experiments. Moreover, clinical trials are underway to validate candidate lncRNAs as biomarkers for the detection and prognosis of high-grade serous ovarian carcinoma (HGSOC) (NCT03738319). In the following sections, we will discuss lncRNAs as potential biomarkers for diagnosis, prognosis, and therapeutic monitoring of OC (**Figure 3**).

### 4.1 Diagnostic and prognostic potential of lncRNAs in OC

The systematic integration of associations between lncRNA and tumor is important for further understanding the underlying molecular mechanisms and exploring lncRNA-based biomarkers and therapies. Accumulating evidence indicates that multiple lncRNAs are dysregulated in OC and are strongly correlated with the degree of tumor differentiation, clinical stage and lymphatic metastasis of patients. Therefore, these lncRNAs may be valuable biomarkers for the diagnosis and prognosis of this neoplasm.

It has been proposed that lncRNA can be utilized as a potential epigenetic-based biomarker for OC. For instance, a recent study reported that methylated lncRNA ARMCX1, ICAM4, LOC134466, PEG3, PYCARD and SGNE1 contributed to diagnosis of serous OC, a panel of the above identified genes possessed an area under the curve (AUC) of 0.98. Moreover, methylation of lncRNA LOC134466 (also known as ZFN300P1), can effectively discriminate cancer samples from normal controls with AUC of 0.72, suggesting it as an independent biomarker of OC (145). Interestingly, metastasis-associated lncRNAs have also been widely mentioned as promising markers of OC prognosis. Another study showed that epigenetic repression of lncRNA ZNF300P1 in OC enhanced the ability of tumor cells to adhere to the peritoneal surface and contributed to the metastatic spread of cancer (146). In addition, another study showed that lncRNA expression varies in OC cells with different metastatic potential. In particular, both lncRNA H19 and MALAT1 were downregulated in highly metastatic OC cells. These lncRNAs may serve as novel diagnostic and therapeutic biomarkers (147).

Evidence for lncRNA as a promising marker for clinical diagnosis and prediction of OC has surfaced. lncRNA Meg3, was under-expressed in OC and was negatively correlated with FIGO stages. More importantly, Meg3 was able to identify benign tumors from OC (AUC, 0.727; 95% CI, 0.600-0.853) and differentiate normal samples from OC tissues (AUC, 0.763; 95% CI, 0.586-0.940) (**Figure 3A**) (72). Additionally, in another independent study, the expression of lncRNA FEZF1-AS1 was significantly increased in both OC specimens and patient serum. Further analysis showed that high level of FEZF1-AS1 was strongly correlated with TNM stage, lymphatic metastasis and OS of patients. In addition, serum levels of FEZF1‐AS1 could distinguish EOC patients from normal controls (AUC, 0.9483, 95% CI, 0.915-0.998). Significantly, based on its high sensitivity and specificity, serum lncRNA FEZF1-AS1 levels were apparently lower in postoperative patients compared with preoperative patients (148). However, studies have also shown that aberrantly expressed lncRNAs can predict tumor resistance to chemotherapy. For example, one study showed that a panel of lncRNAs were aberrantly expressed in PTX-resistant OC tissues and cell lines and correlated with progression-free survival of patients. In addition, combination of all these lncRNA features was more effective in predicting the accuracy of resistance to chemotherapy with a high AUC of 0.93 (95% CI, 0.86-1.00) compared to each individual. More importantly, this group of lncRNAs was characterized as an independent predictor of sensitivity to platinum-based PTX-containing therapy in OC patients. Interestingly, enrichment analysis by a network database showed that insulin secretion-related pathways were involved in the predictive function of these lncRNAs, providing favorable evidence for exploring the mechanism of PTX resistance in OC patients (149).

Moreover, lncRNA LINC00152 was elevated in OC and predicted poor clinical outcomes in patients, including advanced FIGO stage, larger tumor sizes, increased vascular invasion and lymph node metastasis. Furthermore, a combined analysis of the two independent predictors, expression levels of LINC00152 and FIGO stages, could serve as a more sensitive and specific biomarker for OC compared to each individual (150). Additionally, the specific expression of lncRNA HOST2 was dramatically upregulated in OC patients compared to the benign disease controls, contributing to tumor progression (46). While, lncRNA MAGI2-AS3 could exhibit tumor-suppressing effect in HGSOC though interacting with miR-15-5p, miR-374a-5p, and miR-374b-5p. Further studies may focus on uncovering MAGI2-AS3 signatures that could serve as diagnostic and prognostic tools for HGSOC (151). Similarly, lncRNA HOXA11-AS was aberrantly decreased in OC, and one of the exon variants in HOXA11-AS was associated with a reduced risk of serous OC (OR, 0.88; 95% CI, 0.78-1.01) (152). In addition to this, diverse exosomal lncRNAs are correlated with clinicopathologic features of cancer and may act as novel biomarkers (153, 154). Currently, a study demonstrated that exosome lncRNA MALAT1 secreted by tumor cells was upregulated in the serum of patients with OC and that high serum exosome MALAT1 levels were associated with poor outcome in clinical patients. In addition, a prognostic model was constructed using important factors such as serum exosomal MALAT1 levels, FIGO stage, and lymph node metastasis, which exerted a good prediction of 3-year OS in patients with OC (**Figure 3A**) (68). Similarly, serum exosomal lncRNA aHIF was significantly elevated in OC patients compared to healthy controls. Moreover, upregulated exosomal aHIF in serum was positively linked to higher histological grade and FIGO stage, and predicted shorter OS in patients (155). These studies suggest that lncRNAs have potential value in the diagnosis, prognosis, and treatment of OC.

In addition, tumor metabolism and inflammation are distinctive features of cancer and are directly related to the prognosis and severity of the disease (156). Thus, analysis of combined metabolites, inflammatory markers, and lncRNA will facilitate the identification of additional useful serum markers and enable stratified management of OC patients in the future. However, there are some limitations to the utility of lncRNAs as biomarkers due to the fact that multiple studies use paired samples for experiments or benign tumor tissues or normal ovarian tissues as controls to obtain differences in lncRNA expression levels. This does not truly reflect the differences in lncRNA expression between cancer cells and normal cells. Given that different subtypes of OC have intrinsic differences in genetic risk factors, response to chemotherapy and different clinical outcomes (157), studies of lncRNA in multiple histological subtypes should be conducted to lay the foundation for obtaining more specific biomarkers.

### 4.2 Therapeutic potential of lncRNAs in OC

Considering that the dysregulation of lncRNAs is associated with a variety of cellular processes in OC, lncRNAs may be prospective targets for OC therapy. Hence, many ongoing studies are aimed at modulating the production or inhibition of lncRNAs. A growing amount of studies have emphasized that lncRNAs may act as oncogenic drivers or tumor-suppressive roles in OC (43, 49). Currently, studies have focused on the clinical utility of lncRNAs as therapeutic targets of OC (158). Moreover, a comprehensive understanding of the mechanisms of gene knockdown or overexpression may provide new insights into the therapeutic targeting of lncRNAs.

It is well known that RNA interference and antisense oligonucleotides (ASOs) are important components of RNA-based therapeutic strategies (159), which can be fully or incompletely complementary to a large number of heterogeneous transcripts (160–162). Currently, in the study of lncRNAs, RNA interference has been successfully applied in several preclinical models to explore the therapeutic implications for various diseases (163). For instance, lncRNA FEZF1‐AS1 and RHPN1-AS1 are novel oncogenic lncRNAs in OC that can be targeted with small interfering RNA (siRNA) and short hairpin RNA, respectively, to inhibit tumor metastasis and progression (148, 164). Although siRNA and ASOs are often directed against similar targets (165), studies have shown that they differ in achieving gene silencing effects (166). Currently, a study suggests that ASOs may be a potential therapeutic strategy compared to siRNA in inhibiting the malignant progression of OC (**Figure 3C**) (28). Conversely, in the case of lncRNAs with tumor suppressor effects, their functions can be enhanced by overexpression (167).

Actionable ways of determining molecular alterations and selecting targeted treatments are constantly being revolutionized. A recent study found that lncRNA SPOCD1-AS in extracellular vesicles secreted by OC can be delivered to mesothelial cells and interact with G3BP1 protein to trigger the MMT process, thereby promoting peritumoral colonization of tumors. More importantly, G3BP1-interfering peptide was able to block the lncRNA SPOCD1-AS/G3BP1 interaction, thereby reducing peritoneal metastasis *in vivo*. This research provides a latent therapeutic approach for metastatic OC (**Figure 3B**) (168). Furthermore, another study showed that lncRNA PLADE, an exosome-derived lncRNA from HGSOC ascites, exhibited low expressions in tumor tissues and had the potential to synergize with cisplatin to inhibit chemoresistance (169). Similarly, a preclinical study highlighted that rapamycin induces sensitization of OC cells to DDP by increasing lncRNA GAS5 expression, which lays a favorable theoretical foundation for the development of new combination therapies based on chemoresistance (37). Indeed, optimized combinations of RNA-based therapies and drugs have emerged for the treatment of OC resistance. Recently, a study showed that the AURKA/DDX5/TMEM147-AS1/let-7 feedback loop activates lipophagy, thereby maintaining DDP resistance in OC. Importantly, the combination of lncRNA TMEM147-AS1 siRNA and VX-680 effectively enhanced sensitivity of OC to DDP treatment compared to using them separately (158). Similarly, studies have shown that atezolizumab and lncRNA PVT1 inhibitors synergistically suppressed cisplatin resistance in OC cells (83). To date, the nanoparticle-mediated siRNA therapy strategy has made significant breakthroughs in the field of tumor treatment (170). Interestingly, one study demonstrated that a novel nanoparticle delivery platform was able to bring siTWIST into target cells and reverse DDP chemoresistance in an OC model (171). More importantly, a cutting-edge study demonstrated that nanoparticle-mediated siRNA was able to target the lncRNA DANCR and exhibit tumor growth inhibition in a xenograft model of OC (172). This result paves the way for the development of RNA-based delivery systems to target oncogenic lncRNAs for OC therapy. However, translating these technologies into the clinic is challenging because delivery systems need to have good specificity, stability, and low immunogenicity (173, 174). As natural nano-vesicles, exosomes are good carriers for delivering protein and nucleic acid drugs (175). A recent study based on umbilical cord blood-derived M1 macrophage exosomes demonstrated their ability to inhibit platinum resistance in OC by loading DDP. In addition, this exosome carries lncRNA H19, which is involved in the reversal of DDP chemoresistance (**Figure 3D**) (176). This innovative approach could hold promise for the treatment of OC. As academia and industry continue to advance the field of nanoresearch, we expect more lncRNA-targeted drugs to enter the clinic, providing new options for precision medicine for OC patients.

## 5 Conclusions and future directions

The occurrence of OC is a complex process involving multiple genes and multiple steps, and the key molecular events that initiate this complex condition remain to be fully identified. In the above article, we emphasized the role of lncRNAs in the process of OC cell biology, including metastasis, autophagy, apoptosis, ferroptosis, drug resistance, and tumor immunity. In addition, we focus on the multiple molecular mechanisms involved in the regulation of OC tumorigenesis and progression by lncRNAs, such as acting as miRNA sponges, binding to proteins or DNA, etc. Potential clinical applications of lncRNAs in the diagnosis and prognosis of OC are also being explored in search of strategies that can be used as novel therapies for tumors.

Recently, advances in the function and mechanism of lncRNAs paint a daunting picture for therapeutic interventions of OC. However, a number of key questions remain to be elucidated: (i) Does the combination of multiple lncRNA signatures have the potential to serve as a more efficient prognostic marker for OC? Given that the field of lncRNAs in OC is still at an exploratory stage, it is too early to say for sure. Biomarkers are defined as alterations in the composition of fluids or tissues that reflect disease status and progression (177). Apparently, studies have confirmed that a variety of lncRNAs are abnormally expressed in OC and are associated with the malignant biological behavior of tumors and poor prognosis of patients. In addition, the combination of a panel of lncRNA characteristics is more effective in predicting the accuracy of chemotherapy resistance than a single individual (149). (ii) Could lncRNAs in body fluids be candidate biomarkers for OC diagnosis? Liquid biopsies can be used to track disease progression and monitor tumor recurrence with convenience and minimal harm to patients (178). However, there is a relative lack of studies incorporating dysregulated lncRNA in body fluids of OC patients for detection. Therefore, more studies should combine the detection of tumor tissue and body fluid samples, which will help to advance the role of liquid biopsy technology in the diagnosis of OC. (iii) Whether targeting lncRNAs could be served as prospective therapeutic strategy for OC? Currently, lncRNAs play a key role in the diagnosis and prediction of OC, and *in vitro* models have confirmed the carcinostatic effects by targeting them in chemotherapy and targeted therapy. However, the feasibility and safety of delivery systems based on silencing oncogenic lncRNAs in *in vivo* models needs to be further investigated.

Currently, the lack of highly specific and sensitive detection systems and effective therapeutic regimens remains a great challenge for the clinical management of OC. As participants in the important biological processes of OC, novel functions of lncRNAs continue to be investigated. Future studies should focus on elucidating the exact functional mechanisms of lncRNAs, including their involvement in signaling pathways that regulate important OC phenotypes, in order to bridge the gap between basic research and clinical applications, make them new potential targets for cancer therapy, and facilitate early diagnosis as effective biomarkers.

## Author contributions

ZH: Investigation, Formal analysis, Writing – review & editing, Writing – original draft, Software. LY: Writing – review & editing, Data curation. XY: Investigation, Writing – review & editing. CY: Data curation, Validation, Conceptualization, Funding acquisition, Writing – review & editing, Supervision. JL: Validation, Conceptualization, Writing – review & editing, Supervision.
